# A retinoid X receptor partial agonist attenuates pulmonary emphysema and airway inflammation

**DOI:** 10.1186/s12931-018-0963-0

**Published:** 2019-01-03

**Authors:** Daisuke Morichika, Nobuaki Miyahara, Utako Fujii, Akihiko Taniguchi, Naohiro Oda, Satoru Senoo, Mikio Kataoka, Mitsune Tanimoto, Hiroki Kakuta, Katsuyuki Kiura, Yoshinobu Maeda, Arihiko Kanehiro

**Affiliations:** 1 Department of Hematology, Oncology, Allergy and Respiratory Medicine, Okayama University Graduate School of Medicine, Dentistry, and Pharmaceutical Sciences, 2-5-1 Shikata-cho, Okayama, 700-8558 Japan; 2 Department of Pharmacology, Okayama University Graduate School of Medicine, Dentistry, and Pharmaceutical Sciences, Okayama, Japan; 3 Department of Allergy and Respiratory Medicine, Okayama University Hospital, Okayama, Japan; 4 Department of Allergy and Respiratory Medicine, Okayama Rosai Hospital, Okayama, Japan

**Keywords:** Retinoid X receptor, Emphysema, Neutrophilic airway inflammation, Matrix metalloproteinase-9, Anti-oxidant activity, Vascular endothelial growth factor

## Abstract

**Background:**

Retinoid X receptors (RXRs) are members of the nuclear receptor (NR) superfamily that mediate signalling by 9-cis retinoic acid, a vitamin A derivative. RXRs play key roles not only as homodimers but also as heterodimeric partners, e.g., for retinoic acid receptors, vitamin D receptors, and peroxisome proliferator-activated receptors. The NR family may also play important roles in the development of emphysema. However, the role of RXRs in the pathogenesis of emphysema is not well defined.

**Methods:**

We developed a novel RXR partial agonist (NEt-4IB) and investigated its effect and mechanism compared to a full agonist (bexarotene) in a murine model of emphysema. For emphysema induction, BALB/c mice received intraperitoneal cigarette smoke extract (CSE) or intratracheal porcine pancreas elastase (PPE). Treatment with RXR agonists was initiated before or after emphysema induction.

**Results:**

Treatment with NEt-4IB significantly suppressed the increase in static lung compliance and emphysematous changes in CSE-induced emphysema and PPE-induced established and progressive emphysema. NEt-4IB significantly suppressed PPE-induced neutrophilic airway inflammation and the levels of keratinocyte chemoattractant (KC), C-X-C motif ligand5 (CXCL5), interferon (IFN)-γ and IL-17. NEt-4IB also improved the matrix metalloproteinase-9 (MMP-9)/tissue inhibitor of metalloproteinase-1 (TIMP-1) imbalance and the reduced anti-oxidant activity in bronchoalveolar lavage (BAL) fluid. NEt-4IB suppressed PPE-induced vascular endothelial growth factor (VEGF) expression in the airway. Treatment with NEt-4IB and bexarotene significantly suppressed the increase in static lung compliance and emphysematous changes. However, adverse effects of RXR agonists, including hypertriglyceridemia and hepatomegaly, were observed in bexarotene-treated mice but not in NEt-4IB-treated mice.

**Conclusion:**

These data suggest that RXRs play crucial roles in emphysema and airway inflammation, and novel partial RXR agonists could be potential therapeutic strategies for the treatment of PPE- and CSE-induced emphysema.

**Electronic supplementary material:**

The online version of this article (10.1186/s12931-018-0963-0) contains supplementary material, which is available to authorized users.

## Background

Chronic obstructive pulmonary disease (COPD) is characterized by airflow obstruction that is not fully reversible, leading to respiratory insufficiency and functional disability [1]. Existing therapeutic options for COPD can reduce symptoms and the frequency and severity of exacerbations. Nonetheless, these therapies are not able to effectively preserve lung function or completely palliate symptoms in COPD [1]. Therefore, new anti-inflammatory therapeutic strategies are required.

Recent investigations have demonstrated that nuclear receptors (NRs) are related to the development of COPD. Mice lacking peroxisome proliferator-activated receptor γ (PPARγ) were reported to exhibit enhanced cigarette smoke (CS)-induced airway inflammation and emphysematous changes in the lungs [2]. Rosiglitazone, which is an agonist of PPARγ, attenuated CS-induced airway inflammation and emphysematous changes in the lungs [3]. Gene expression of liver X receptor (LXR) in the lungs was increased in COPD patients, and an LXR agonist exerted anti-inflammatory effects on C-X-C motif ligand 10 (CXCL10), C-C motif ligand 5 (CCL5), and IL-10 production by alveolar macrophages [4]. Retinoid X receptors (RXRs) are NRs that control gene transcription in a manner dependent on ligand binding [5] and act as homodimers or heterodimers with PPARs, LXRs, and other NRs [6–8]. These data suggest that RXRs may be potential targets for the prevention and management of COPD. The efficacy of RXR agonists for treatment of several diseases has been reported. For example, the RXR full agonist bexarotene has been used for the treatment of cutaneous T cell lymphoma [9] and has been reported to have therapeutic potential for treatment of animal models of type-2 diabetes [10], Alzheimer’s disease [11], and Parkinson’s disease [12]. We previously reported that a new RXR full agonist, NEt-TMN, has the potential to reduce adverse effects while retaining desired activities [13]. However, RXR full agonists including NEt-TMN and bexarotene also induce various adverse events, such as weight gain, hepatomegaly, and blood triglyceride elevation, when used to treat underlying diseases [13].

Partial agonists have only partial efficacy on receptors relative to full agonists and exert both agonistic and antagonistic effects. Clinically, partial agonists can be used to activate receptors to give a desired submaximal response and prevent overstimulation or prolonged activation of receptors, which could cause adverse events. It was reported that activation of LXRα/RXRα induced increased levels of triglycerides in the serum [14]. To avoid severe adverse events, we planned to induce moderate activation of RXRs sufficient to regulate airway inflammation and focused on the production of novel RXR partial agonists with lower maximum capacities to activate RXRs than that of full RXR agonists. We finally developed the innovative RXR partial agonist NEt-4IB, 55% of the activity of a full agonist in a luciferase reporter assay [15]. In addition, we previously reported that long-term administration of NEt-4IB induced no significant adverse events [15].

In the present study, to determine whether RXRs play important roles in the development of emphysema and whether novel RXR partial agonists may be promising options for the treatment of emphysema, we investigated the therapeutic potential and adverse effects of these molecules on emphysema development and airway inflammation in a murine model of emphysema.

## Methods

### Animals

Female BALB/c mice at 8 weeks of age were purchased from Charles River Japan, Inc. (Yokohama, Japan). All experimental animals used in the present study were housed under a protocol approved by the Institutional Animal Care and Use Committee of Okayama University Medical School (Okayama, Japan).

### COPD induction by cigarette smoke extract

For the preparation of cigarette smoke extract (CSE), 3R4F reference cigarettes were purchased from the University of Kentucky. One non-filtered cigarette was burned, and the smoke was passed through 8 ml PBS by connecting to a vacuum pump with a constant air flow of 2 L/min. The extract was used when the pH was between 6.2 and 6.4, and the OD at 320 nm was between 1.600 and 1.900. The extract was prepared fresh for each experiment and used for administration after adjusting the pH to between 7.00 and 7.40 and filtering through a 0.2-μm-pore filter to remove particles and bacteria. Mice were anaesthetized with isoflurane for administration of CSE. The experimental mice received 400 μl CSE intraperitoneally on days 0, 7, 14, and 21. The control mice received 400 μl phosphate buffered saline (PBS) at the same time points.

### COPD induction by porcine pancreas elastase

Mice were anaesthetized with isoflurane for the administration of porcine pancreas elastase (PPE) (Sigma-Aldrich, St. Louis, MO, USA). The experimental mice received 50 U/kg PPE dissolved in 40 μl PBS via the trachea. The control mice received 40 μl PBS at the same time points.

### Administration of the novel RXR partial agonist and bexarotene

The mice orally received a novel RXR partial agonist (0.005, 0.015%, or 0.05% NEt-4IB; see Additional file 1) or a full RXR agonist (0.015% bexarotene). In detail, mice were fed standard diet containing RXR agonist (mixed feed) with free eating system by mice. The control mice received vehicle. The changes of the weight of feed in each cage were measured every day or ever week according to the study protocol.

### Determination of static lung compliance

A flexiVent small-animal ventilator (SCIREQ, Montreal, PQ, Canada) was used to assess static lung compliance (Cst), which is a measurement of the elasticity of the lungs. It was calculated from the pressure volume curves using flexiVent software (version 5.0) [16], as previously described. In brief, the mice were anaesthetized with 150 mg/kg ketamine and 10 mg/kg xylazine (Kyoritsu Seiyaku, Tokyo, Japan) by intraperitoneal injection. The mice were tracheostomized with a 5-mm section of metallic tubing (18 G cannula) and ventilated at 150 breaths/min with a tidal volume of 10 ml/kg and a positive end-expiratory pressure of 3 cmH_2_O, and Cst was then measured.

### Bronchoalveolar lavage fluid

Lungs were lavaged with Hanks’ balanced salt solution via the tracheal tube (2 × 1 ml, 37 °C), and the number of cells in the bronchoalveolar lavage (BAL) fluid was counted. Cytospin slides were stained and differentiated in a blinded fashion by counting at least 200 cells under light microscopy.

### Lung histology and morphometric measurements of air space size

The left lung was inflated by intratracheal instillation with 10% formalin at a static pressure of 20 cmH_2_O and fixed in 10% formalin. The tissue was then embedded in paraffin, and 2-μm thick sections were stained with haematoxylin and eosin (H&E). Air space enlargement was quantified by assessing the mean linear intercept (Lm) and the destructive index (DI). Ten of tissue image fields were randomly selected from all parts of the whole lung at the height of the hilum from each mouse. The Lm, which represents the average size of the alveoli, was calculated by counting lines of a defined length, as previously described [17]. In detail, we put 10 traversed lines provided at regular intervals on the lung section. A total number of intercepts with the lung structures on each traversed line are counted. A total 1000 lines per each mouse lung were evaluated. Lm is calculated as the ratio between the sum of the length of traverse lines and the sum of all the intercepts. The DI, which represents the proportion of destroyed alveoli among the total alveoli, was determined by dividing the number of identified destroyed alveoli. We put 50 counting points on each image. Only point putting on alveolar and duct space were counted. If a counting point was surrounded by normal alveolar or alveolar duct, they are considered as normal [18, 19]. Destruction was counted when there were more than two alveolar wall defects, when there were more than two intraluminal parenchymal rags in alveolar duct, when clearly abnormal morphology, or when classic emphysematous changes. More than 3000 alveoli randomly were counted in each group. DI was represented as percentage of the destructive alveoli as a fraction of total number of alveoli.

### Cytokine and chemokine measurements

The cytokine levels in the BAL fluid and in the supernatants of homogenized lungs were measured by ELISA, as previously described [20]. For preparation of lung homogenates, lung tissue was frozen at − 70 °C immediately after euthanasia. The lung tissue was mixed with a PBS-0.1% Triton-X100 solution containing proteinase inhibitors at a 1:2.5 ratio of weight per volume (Sigma-Aldrich). The specimens were homogenized and then centrifuged at 21,480 g for 30 min. The supernatants were frozen at − 80 °C until analysis. All cytokine and chemokine ELISAs were performed according to the manufacturer’s instructions. The limits of detection were 2 pg/ml for keratinocyte chemoattractant (KC), 1.74 pg/ml for CXCL5, 2 pg/ml for interferon (IFN) -γ, 5 pg/ml for IL-17A, 1.8 pg/ml for IL-6, 4.8 pg/ml for IL-1β, 7.2 pg/ml for tumour necrosis factor (TNF)-α, 3 pg/ml for vascular endothelial growth factor (VEGF), 0.014 ng/ml for matrix metalloproteinase-9 (MMP-9), 3.5 pg/ml for tissue inhibitor of metaroproteiase-1 (TIMP-1) (R&D Systems, Minneapolis, MN, USA), and 40 pg/ml for perforin (Diaclene, France).

### Measurement of anti-oxidant activity in BAL fluid and serum

The anti-oxidant activity in the BAL fluid and the serum was determined by the bio-anti-oxidant power (BAP) test using a Free Radical Elective Evaluation FRS4 system (Diacron International, Grosseto, Italy), as previously described [18].

### Measurement of serum parameters

Triglyceride (TG), glucose, aspartate transaminase (AST), alanine aminotransferase (ALT), alkaline phosphatase (ALP), amylase (AMY), and leucine aminopeptidase (LAP) levels were measured using a Fuji Dry Chem system (Dry Chem 4000 V; Fuji Medical Co., Tokyo, Japan).

### Statistical analysis

All results were described as the means ± standard error of the means (SEM). Statistical significance among all groups was detected by using a one-way ANOVA. Pairs of groups of samples distributed parametrically were compared by unpaired two-tailed Student’s t-tests, and samples distributed nonparametrically were compared by the Mann-Whitney U test. Significance was assumed at *P* values < 0.05.

## Results

### The optimal dosage of the novel RXR partial agonist NEt-4IB in a murine model

We assessed the dose-related effects of the novel RXR partial agonist NEt-4IB in a murine model of emphysema to determine the optimal dosage of NEt-4IB in an emphysema model (see Additional files 1, 2). These results suggested that the optimal concentration of NEt-4IB in our murine emphysema model was 0.015%; this concentration has therapeutic efficacy without inducing adverse effects and is consistent with our recent study in a murine model of asthma [20].

### Treatment with the novel RXR partial agonist prevents CSE-induced emphysematous changes

**Fig. 1:**
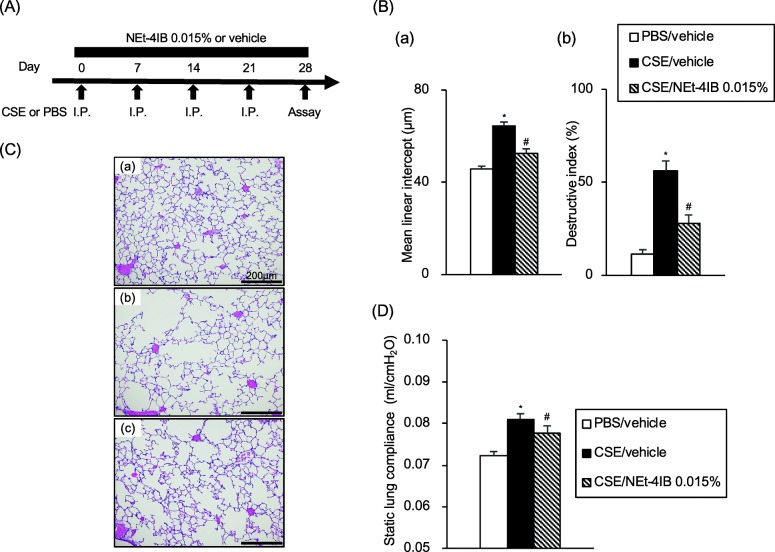
Treatment with a novel RXR partial agonist prevents CSE-induced emphysematous changes. **a** Experimental protocol. **b** (*a*) Lm values, (*b*) DI values. **c** Representative photographs of H&E-stained lung tissue (magnification: X200) (*a*) PBS/vehicle, (*b*) CSE/vehicle, (*c*) CSE/NEt-4IB 0.015%. (D) Cst values. The results for each group are expressed as the means ± SEM. This experiment was started with 7 mice in PBS/vehicle, and 11 mice in CSE/vehicle, PPE/NEt-4IB 0.015%. No mice were died during the experimental period. * Significant differences (*P* < 0.05) between PBS/vehicle and CSE/vehicle. # Significant differences (*P* < 0.05) between CSE/vehicle and CSE/NEt-4IB 0.005%. CSE, cigarette smoke extract

We assessed the efficacy of the novel RXR partial agonist in CSE-induced emphysema (Fig. 1a). Intraperitoneal administration of CSE to vehicle-treated mice significantly increased Lm and DI values on day 28 compared to PBS/vehicle mice. Treatment with 0.015% NEt-4IB significantly suppressed the increases in Lm and DI values compared to the increases in CSE/vehicle mice (Fig. 1b). Representative light microscopic photographs of lung sections are shown in Fig. 1c. Additionally, the Cst values in CSE/vehicle mice on day 28 were significantly increased compared to those of PBS/vehicle mice, and treatment with NEt-4IB significantly attenuated the increase in Cst compared to that observed in CSE/vehicle mice (Fig. 1d).

### Treatment with NEt-4IB suppresses established and progressive emphysematous changes

**Fig. 2:**
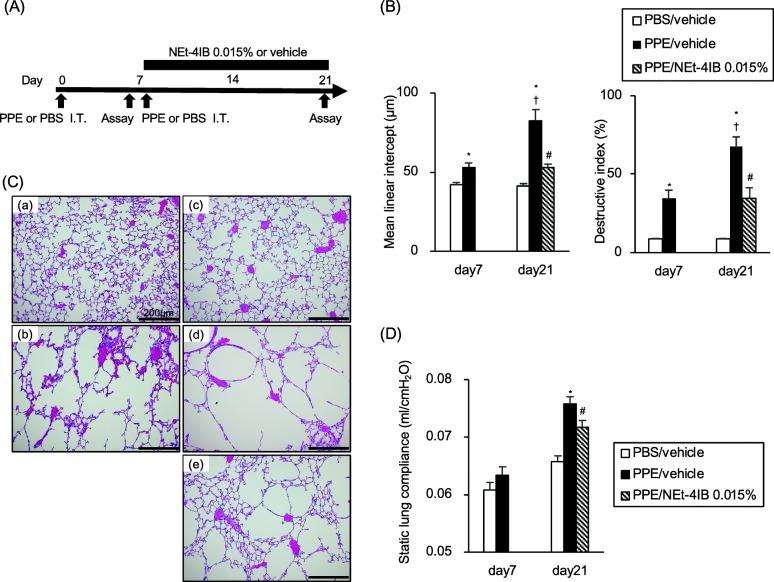
Treatment with NEt-4IB suppresses established and progressive emphysematous changes. **a** Experimental protocol. **b** (*a*) Lm values, (*b*) DI values. **c** Representative photographs of H&E-stained lung tissue (magnification: X200) (*a*) PBS/vehicle on day 7, (*b*) PPE/vehicle on day 7, (*c*) PBS/vehicle on day 21, (*d*) PPE/vehicle on day 21, (*e*) PPE/NEt-4IB 0.015% on day 21. **d** Cst values. The results for each group are expressed as the means ± SEM. This experiment was started with 8 mice in each group, and no mice were died during the experimental period. * Significant differences (*P* < 0.05) between PBS/vehicle and PPE/vehicle. # Significant differences (*P* < 0.05) between PPE/vehicle and PPE/NEt-4IB 0.015% † Significant differences (*P* < 0.05) between PPE/vehicle on day 7 and PPE/vehicle on day 21

Next, we assessed whether NEt-4IB could prevent the progression of emphysematous changes (Fig. 2a). On the seventh day after first intratracheal administration of PPE, emphysematous changes in the lungs were already observed in PPE-treated mice. Lm and DI were significantly increased in PPE/vehicle mice compared with PBS/vehicle mice on day 7 (Fig. 2b). To further evaluate the progression of emphysematous changes, PPE/vehicle mice were administered a second dose of PPE on day 7. This additional PPE instillation significantly promoted the increases in Lm and DI compared to those observed in both PPE/vehicle mice on day 7 and PBS/vehicle mice on day 21. Interestingly, treatment with NEt-4IB from days 7 to 21 significantly suppressed the PPE-induced increases in Lm and DI compared to those observed in PPE/vehicle mice on day 21 (Fig. 2b). Representative light microscopic photographs of lung sections are shown in Fig. 2c. According to the pulmonary function test, the Cst values in PPE/vehicle mice on day 7 did not significantly increase compared to those of PBS/vehicle mice. However, after the second PPE instillation, the Cst values in PPE/vehicle mice on day 21 did significantly increase compared to those of PBS/vehicle mice, and treatment with NEt-4IB significantly suppressed this increase in Cst value compared to the increase observed in PPE/vehicle mice (Fig. 2d).

### Treatment with NEt-4IB attenuates PPE-induced airway inflammation

**Fig. 3:**
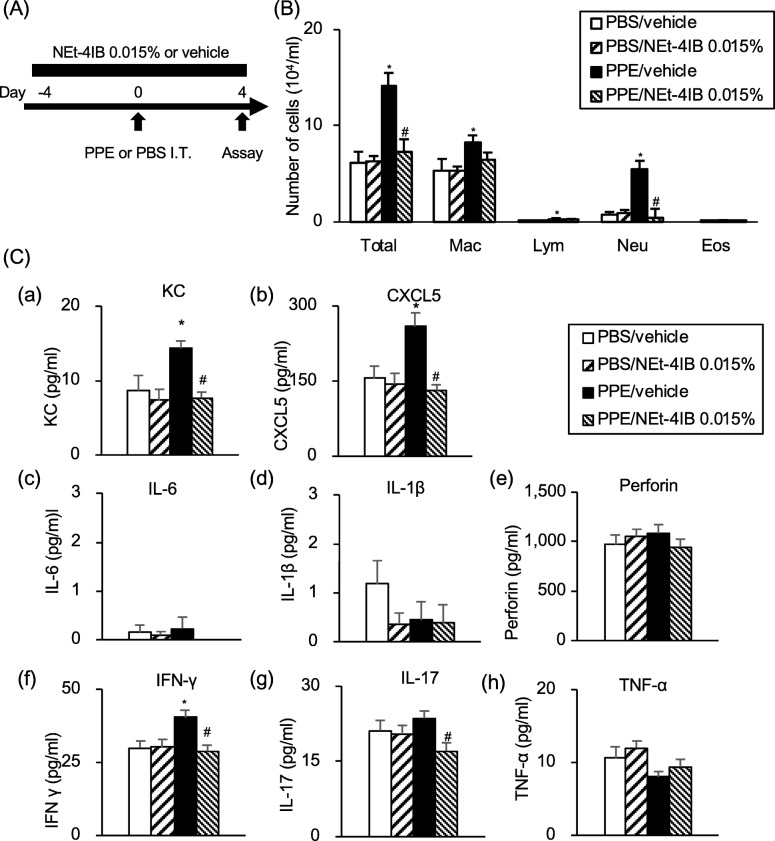
Treatment with NEt-4IB attenuates PPE-induced airway inflammation. **a** Experimental protocol. **b** Cell composition in BAL fluid. **c** (*a*) KC, (*b*) CXCL5 (*c*) IL-6, (*d*) IL-1β in BAL fluid, and (*e*) perforin, (*f*) IFN-γ, (*g*) IL-17, and (*h*) TNF-α in the lungs. The results for each group are expressed as the means ± SEM. This experiment was started with 10 mice in each group, and no mice were died during the experimental period. * Significant differences (*P* < 0.05) between PBS/vehicle and PPE/vehicle. # Significant differences (*P* < 0.05) between PPE/vehicle and PPE/NEt-4IB 0.015%

To determine whether treatment with NEt-4IB can affect inflammatory changes in the lungs, we assessed airway inflammation in BAL fluid or lung tissue in a murine model of PPE-induced emphysema (Fig. 3a). The numbers of macrophages, lymphocytes, and neutrophils in BAL fluid were significantly increased in PPE/vehicle mice compared to PBS/vehicle mice. Treatment with NEt-4IB significantly reduced the numbers of total cells and neutrophils in BAL fluid compared to those seen in PPE/vehicle mice (Fig. 3b). The levels of KC and CXCL5 in BAL fluid and IFN-γ in lung tissue were also significantly increased in PPE/vehicle mice compared to PBS/vehicle mice (Fig. 3c *a*, *b*, *f*). Treatment with NEt-4IB significantly reduced the levels of KC and CXCL5 in BAL fluid and IFN-γ and IL-17 in lung tissue compared to levels in PPE/vehicle mice (Fig. 3c *a*, *b*, *f*, *g*). PPE did not significantly increase the level of perforin in the lung compared to that of PBS/vehicle mice; nevertheless, treatment with NEt-4IB tended to reduce this augmentation (Fig. 3c *e*). IL-6 and IL-1β levels in the BAL fluid were below the threshold of measurement sensitivity (Fig. 3c *c*, *d*), and there was no significant difference in the levels of TNF-α in lung tissue (Fig. 3c *h*).

### Treatment with NEt-4IB improves the PPE-induced proteinase/anti-proteinase imbalance in BAL fluid and the reduction of anti-oxidant activity in the airway

**Fig. 4:**
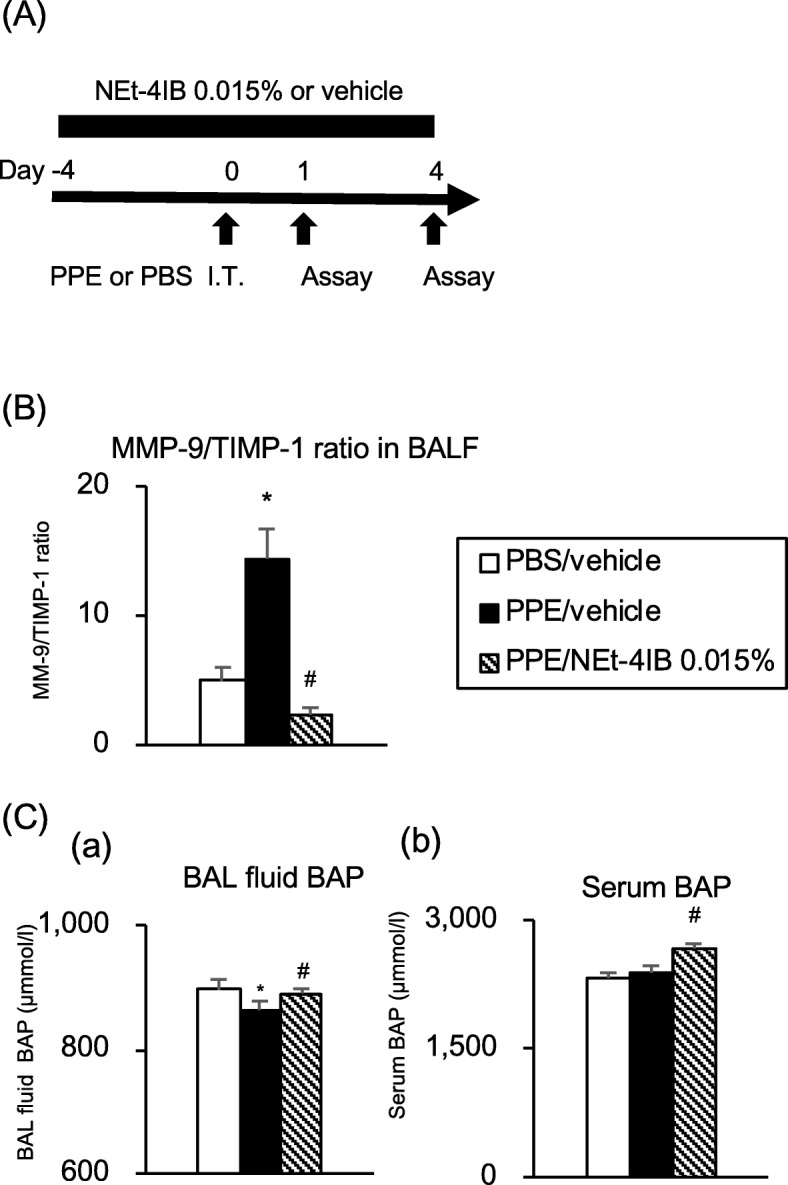
Treatment with NEt-4IB improves the PPE-induced proteinase/anti-proteinase imbalance and impairment of anti-oxidant activity. **a** Experimental protocol. **b** MMP-9/TIMP-2 ratio in BAL fluid on day 4. **c** Anti-oxidant activity measured by the BAP test in BAL fluid (*a*) and in serum (*b*) on day 1. The results for each group are expressed as the means ± SEM. This experiment was started with 10 mice in each group, and no mice were died during the experimental period. * Significant differences (*P* < 0.05) between PBS/vehicle and PPE/vehicle. # Significant differences (*P* < 0.05) between PPE/vehicle and PPE/NEt-4IB 0.015%. BAP, bio-anti-oxidant power

A proteinase/anti-proteinase imbalance has been implicated in COPD pathogenesis in both experimental models and humans. We assessed the MMP-9/TIMP-1 balance on day 4 and anti-oxidant activity on day 1 (Fig. 4a). The MMP-9/TIMP-1 ratio in the BAL fluid was significantly increased in PPE/vehicle mice compared with PBS/vehicle mice, but treatment with NEt-4IB significantly decreased the MMP-9/TIMP-1 ratio (Fig. 4b). Oxidative stress has also been associated with the pathogenesis of COPD in both experimental models and humans. Therefore, we assessed anti-oxidant activity in BAL fluid and serum on day 1. Anti-oxidant activity in the BAL fluid was significantly reduced in PPE/vehicle mice compared to PBS/vehicle mice, and treatment with NEt-4IB significantly improved the reduction of anti-oxidant activity in BAL fluid. This anti-oxidant activity in the serum was not reduced by PPE instillation, although treatment with NEt-4IB significantly increased the anti-oxidant activity compared to that in PBS/vehicle and PPE/vehicle mice (Fig. 4c).

### Treatment with NEt-4IB suppressed PPE-induced VEGF expression in the airway

**Fig. 5:**
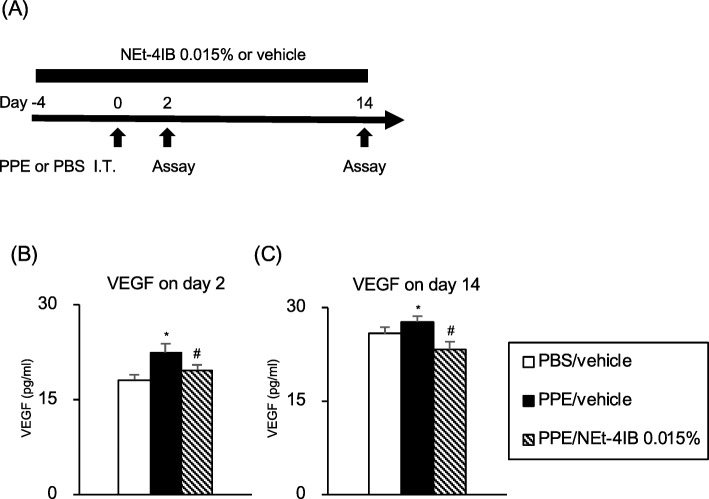
Treatment with NEt-4IB supresses PPE-induced VEGF expression in the airway. **a** Experimental protocol. **b** VEGF in BAL fluid on day 2, (**c**) VEGF in BAL fluid on day 14. The results for each group are expressed as the means ± SEM. This experiment was started with 8 mice in each group, and no mice were died during the experimental period. * Significant differences (*P* < 0.05) between PBS/vehicle and PPE/vehicle. # Significant differences (*P* < 0.05) between PPE/vehicle and PPE/NEt-4IB 0.015%

Angiogenesis-induced airway remodelling plays an important role in the pathogenesis and the development of COPD [21]. We assessed the levels of VEGF in the acute phase on day 2 and the late phase on day 14 in our mouse model (Fig. 5a), and the levels of VEGF significantly increased in PPE/vehicle mice compared to PBS/vehicle mice. Treatment with NEt-4IB significantly attenuated the increase of VEGF levels in PPE/NEt-4IB mice compared to PPE/vehicle mice on both day 2 and day 14 (Fig. 5b, c).

### Comparison of the effects of RXR partial agonist (NEt-4IB) and full agonist (bexarotene) on Lm, DI and Cst

**Fig. 6:**
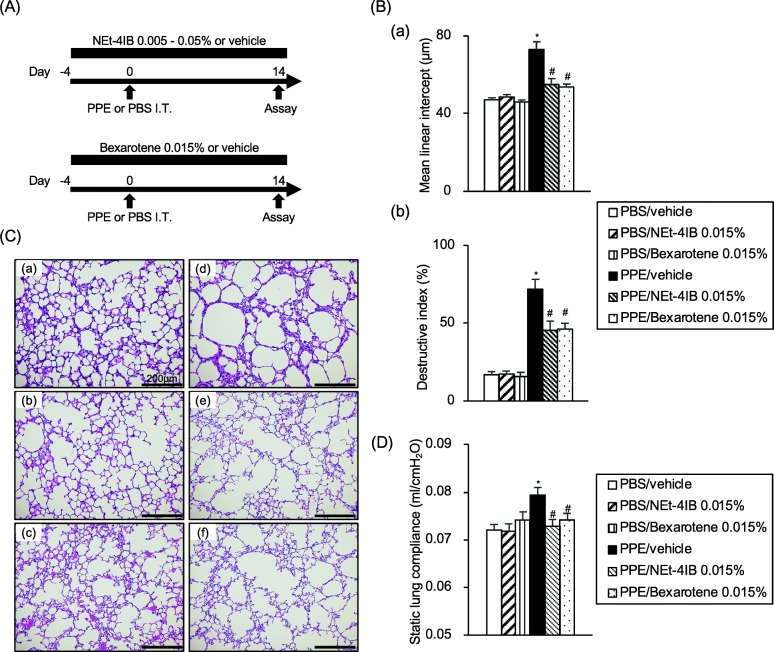
Comparison of the effects of the RXR partial agonist NEt-4IB and the full agonist bexarotene on Lm, DI and Cst. **a** Experimental protocol. **b** (*a*) Lm values, (*b*) DI values. **c** Representative photographs of H&E-stained lung tissue (magnification: X200) (*a*) PBS/vehicle, (*b*) PBS/NEt-4IB 0.015%, (*c*) PBS/bexarotene 0.015%, (*d*) PPE/vehicle, (*e*) PPE/NEt-4IB 0.015%, (*f*) PPE/bexarotene 0.015%. **d** Cst values. The results for each group are expressed as the means ± SEM. This experiment was started with 8 mice in PBS/vehicle, PBS/NEt-4IB 0.015%, PBS/bexarotene 0.015%, and 10 mice in PPE/vehicle, PPE/NEt-4IB 0.015%, PPE/bexarotene 0.015%. No mice were died during the experimental period. * Significant differences (*P* < 0.05) between PBS/vehicle and PPE/vehicle. # Significant differences (*P* < 0.05) between PPE/vehicle and PPE/NEt-4IB 0.015% or PPE/bexarotene 0.015%

We compared the efficacy of the novel RXR partial agonist (NEt-4IB 0.015%) with that of the RXR full agonist (bexarotene 0.015%) (Fig. 6a). Treatment of PPE-treated mice with bexarotene (PPE/bexarotene 0.015%) significantly suppressed the increases in Lm and DI values observed in PPE/vehicle mice (Fig. 6b). Representative light microscopic photographs of lung sections are shown in (Fig. 6c). According to the pulmonary function test, treatment with bexarotene and NEt-4IB significantly suppressed the PPE-induced increase in static lung compliance observed in PPE/vehicle mice (Fig. 6d). There were no significant differences in Lm, DI, and Cst values between PPE/NEt-4IB 0.015% mice and PPE/bexarotene 0.015% mice. These data suggest that RXR agonists have the potential to attenuate the development of emphysema.

### Comparison of adverse events associated with NEt-4IB and bexarotene regarding the liver weight to body weight ratio and serum parameters

The amount of feed intake per day per mouse was not significantly different in each group (Additional file 2). To assess the adverse effects associated with RXR agonists, we measured the body and liver weight (Additional file 2) and the liver weight to body weight (L/BW) ratio and serum parameters (Table 1, Additional file 1). Treatment with NEt-4IB significantly improved the PPE-induced suppression of the ratio of BW change in a dose-dependent manner (Additional file 1D), but liver weight in PPE/NEt-4IB 0.05% mice significantly increased compared to PPE/vehicle mice. Furthermore, liver weight of PPE/bexarotene 0.015% mice was significantly higher than that of PPE/NEt-4IB 0.05% mice (Additional file 2). The L/BW ratio in PPE/NEt-4IB 0.015% mice was slightly increased compared to that of PPE/vehicle mice, but there was no significant difference between PBS/vehicle and PPE/NEt-4IB 0.015% mice. In contrast, the L/BW ratio in PPE/bexarotene 0.015% mice was significantly higher than in PBS/vehicle, PPE/vehicle, and PPE/NEt-4IB 0.015% mice (Table 1). Regarding serum parameters, the level of TGs in PPE/vehicle mice was significantly lower than that of PBS/vehicle mice. Treatment with NEt-4IB 0.015% significantly improved the PPE-induced suppression of TG, and there was no significant difference between PBS/vehicle and PPE/NEt-4IB 0.015% mice. In mice treated with bexarotene 0.015%, the TG level was significantly increased compared to those of PBS/vehicle and PPE/NEt-4IB 0.015% mice. Treatment with NEt-4IB 0.015% did not affect the levels of glucose, AST, ALT, ALP, LAP, or AMY in blood serum, which were measured to evaluate liver and pancreas damage. In contrast, serum levels of ALP and AMY in bexarotene-treated mice were significantly increased compared to those of PPE/vehicle mice, and serum levels of ALP in bexarotene-treated mice were significantly increased compared to those of PBS/vehicle and PPE/NEt-4IB 0.015% mice (Table 1).

**Table 1:** The L/BW ratio and serum parameters as adverse effects of RXR agonists

	PBS/vehicle	PPE/vehicle	PPE/NEt-4IB 0.015%	PPE/Bexarotene 0.015%
L/BW ratio	0.050 ± 0.002	0.049 ± 0.001	0.053 ± 0.001^#^	0.073 ± 0.002^*, ^^#, ##^
TG (mg/dl)	173 ± 12	140 ± 11*	177 ± 11^#^	255 ± 17^*, ^^#, ##^
Glucose (mg/dl)	482 ± 23	479 ± 21	565 ± 35	492 ± 30
AST (IU/L)	125 ± 13	151 ± 12	153 ± 19	123 ± 12
ALT (IU/L)	56 ± 9	66 ± 7	54 ± 5	31 ± 4^*, ^^#, ##^
ALP (IU/L)	275 ± 10	303 ± 9	296 ± 6	406 ± 11^*, ^^#, ##^
LAP (IU/L)	54 ± 2	51 ± 1	53 ± 1	52 ± 1
AMY (IU/L)	5352 ± 509	4608 ± 276	5155 ± 445	6049 ± 204^#^

*Significant differences (*P* < 0.05) between PBS/vehicle and PPE/vehicle or PPE/Bexarotene 0.015%. ^#^Significant differences (*P* < 0.05) between PPE/vehicle and PPE/NEt-4IB 0.015% or PPE/bexarotene 0.015%. ^##^Significant differences (*P* < 0.05) between PPE/NEt-4IB 0.015% and PPE/bexarotene 0.015%

## Discussion

In the present study, we demonstrated for the first time that RXRs may have important roles in the development of emphysema, and our novel RXR partial agonist NEt-4IB exhibited therapeutic potential for use in a murine model of emphysema. Our data clearly showed that treatment with NEt-4IB significantly suppressed histologic emphysematous changes and the increase in Cst values, as supported not only by the attenuation of neutrophil accumulation and expression of neutrophilic chemotactic factors but also by improvements in the proteinase/anti-proteinase imbalance and in the reduced anti-oxidant activity. Furthermore, treatment with NEt-4IB significantly attenuated the increased VEGF levels in emphysematous mice, which is an important factor for the development of airway remodelling, and suppressed both established and progressive emphysematous changes. These data acquired for both PPE- and CSE-induced emphysema models suggest that the novel RXR partial agonist NEt-4IB has the potential to attenuate the development of emphysema.

RXR, retinoic acid receptor (RAR), and the associated ligands play critical roles in the generation of the lungs. High concentrations of retinoid binding protein and retinoic acid binding protein are observed during the perinatal period [22]: furthermore, RAR and RXR play important roles in the generation of the lungs and in septation of the alveoli [23]. Many studies in animals and humans have been conducted using retinol and retinoid derivatives, which activate RAR and RXR. Am80, which is an RAR alpha agonist, had therapeutic effects in a murine model of emphysema [24]. In contrast, all-trans retinoic acid improved the diffusing capacity of the lung carbon monoxide (DLCO) and CT density score in patients with emphysema with some adverse effects, although no clinical benefit was reported [25]. Palovarotene, a selective RARγ agonist, failed to induce clinical effects in patients with moderate to severe COPD [26]. These data suggest that RAR agonists alone may not have sufficient therapeutic potential to prevent and improve emphysema. RXRs function not only as homodimers but also as permissive heterodimers with PPAR and LXR [5]. Therefore, RXR agonists may exert potential effects on RXR/RXR, PPARγ/RXR and LXR/RXR.

Bexarotene, which is an RXR full agonist, is known to be effective against cutaneous T cell lymphoma in humans [9] and has been reported to have therapeutic potential in animal models of type-2 diabetes [10], Alzheimer’s disease [11], and Parkinson’s disease [12]. However, bexarotene has various strong adverse events, such as weight gain, hepatomegaly, hypertriglyceridemia, pancreatitis, and hypothyroidism [13]. Therefore, we developed a novel RXR partial agonist, NEt-4IB, that reduced adverse events in these assays, in contrast to full agonists, even after 28 consecutive days of administration [15]. Partial agonists are drugs that bind to and activate a receptor but exert only partial effects on the receptor relative to a full agonist. We believe that an important key property of partial agonists is that they display both agonistic and antagonistic effects. Weak partial agonists are compounds that are able to produce only a small percentage of the total response produced by an agonist and that act predominantly as antagonists. Strong partial agonists may come close to mimicking the maximum effects of a full agonist and may display only weak antagonistic ability. Clinically, partial agonists can be used to activate receptors to give a desired submaximal response and prevent overstimulation of receptors, which could result in adverse events. NEt-4IB was found to have a maximum RXR activation efficacy of 55% compared with full agonists based on a luciferase reporter gene assay [15]. To assess the clinical advantages of NEt-4IB compared to bexarotene, we compared the effects of NEt-4IB and bexarotene in a murine model of PPE-induced emphysema. Treatment with bexarotene attenuated PPE-induced emphysematous changes to the same extent as treatment with NEt-4IB, but bexarotene induced less favourable side effect profile than NEt-4IB in the assays studied. We found that administration of bexarotene induced hepatomegaly, hypertriglyceridemia, and ALP and AMY elevation, which were not observed upon treatment with NEt-4IB. Although we did not evaluate the dose-related effects of bexarotene, the human equivalent dose (HED) [27] of bexarotene in the mice in our study (HED 1.5 mg/kg) corresponded to approximately 20% of the dose of bexarotene used to clinically treat cutaneous T cell lymphoma (300 mg/m^2^). It was demonstrated that even a low dose of bexarotene induced hypertriglyceridemia and could cause pancreatitis [28]. These data suggest that NEt-4IB treatment has promising therapeutic potential without inducing the severe adverse events associated with bexarotene treatment.

Accumulation of inflammatory cells, including neutrophils, macrophages, and CD8^+^ T cells, a proteinase/anti-proteinase imbalance, apoptosis, and oxidative stress may play important roles in the pathogenesis of COPD [29]. Neutrophils, which secrete MMP-9, cathepsin G, and neutrophil elastase, are a predictor of the disease severity of the emphysematous component of COPD [29, 30]. Human MMP-9 induced emphysema in a murine model [31], and an MMP-9/TIMP-1 imbalance was observed in COPD patients with exacerbations [32]. According to these data, controlling neutrophilic inflammation may prevent the progression of emphysema. Various nuclear receptor agonists were reported to control neutrophil accumulation and the expression of MMP-9, and agonists of PPARγ and LXR exerted some anti-neutrophilic inflammatory and protective effects in the development of emphysema in a murine model [3]. Bexarotene reduced the levels of MMPs and increased the levels of TIMPs in human alveolar adenocarcinoma cells [33] and in a rat model of cerebral ischaemia [34]. Our data show that treatment with NEt-4IB significantly reduced the levels of IL-17, KC and CXCL5 in BAL fluid and the levels of IFN-γ in lung tissue, consequently reducing neutrophil accumulation and improving the MMP-9/TIMP-1 imbalance in the airways. In the present study, the levels of IL-6, IL-1β and TNF-α did not increase after PPE-instillation. The cause of this is likely that 50 U/kg PPE may not be sufficient for inducing these cytokines in our murine model of emphysema. While eosinophilic inflammation in the airway of COPD patients has recently attracted significant clinical attention, we previously demonstrated that NEt-4IB significantly suppressed airway hyperresponsiveness and eosinophil accumulation in the airways [20] and attenuated the levels of IL-5, IL-13, and NO in BAL fluid. NEt-4IB also reduced the numbers of CD4^+^ and CD8^+^ T cells, TNF-α levels, and nuclear factor-κB (NF-κB) expression in lung tissue in a murine model of allergic asthma [20]. Taken together, these findings indicate that the novel RXR partial agonist NEt-4IB regulates airway inflammation and prevents the progression of various phenotypes of emphysema.

Oxidative stress plays important roles in the pathogenesis of COPD [35]. Smoke from cigarettes, wood, and plastic contains high concentrations of free radicals and other oxidants [35, 36]. Oxidative stress is increased in smokers and patients with acute exacerbations of COPD [37], and the levels of oxidants in exhaled breath condensate are increased in patients with COPD [38]. PPARγ regulates gene transcription, acting as a heterodimer with RXR, and PPARγ/RXR promotes Nrf2, which regulates the transcription of genes, including the anti-oxidant response element [39]. In the present study, treatment with NEt-4IB significantly increased anti-oxidant activity in BAL fluid compared to PPE/vehicle mice. There was no significant difference in the anti-oxidant activity in the serum between PBS/vehicle and PPE/vehicle. Although intratracheal administration of 50 U/kg of PPE could not induce systemic inflammation which could reduce anti-oxidant activity in the serum, treatment with NEt-4IB significantly increased the anti-oxidant activity compared to PPE/vehicle mice. These data suggest that NEt-4IB may have the potential to induce anti-oxidant activity by the anti-oxidant response element activation via PPARγ/RXR.

Chronic airway inflammation results in airway and vascular remodelling in COPD as it does in asthma [21]. The air flow limitation caused by airway remodelling is one of the important factors leading to the pathogenesis of COPD [40]. VEGF could have important roles in airway inflammation, tissue remodelling, and angiogenesis in COPD [21]. VEGF levels were significantly higher in the sputum of COPD patients compared to non-smokers [41] . The expression of VEGF in the bronchiolar epithelium, bronchiolar vascular smooth muscle cells, and bronchiolar airway smooth muscle cells was significantly increased in COPD patients compared to non-COPD patients [42]. In addition, the increased VEGF levels were inversely correlated with the values of FEV1 in COPD patients [42]. Furthermore, the blockade of VEGF significantly suppressed small airway remodelling in a rat model of COPD [43]. These data suggest that suppression of VEGF in the airway may be a therapeutic strategy to prevent the progression of COPD. Although few data have been reported regarding the relationship between angiogenesis and MMPs in the pathogenesis of COPD, the levels of MMP-9 and VEGF in sputum were significantly higher in asthma patients compared to non-asthma patients, and the levels of MMP-9 were significantly correlated with the levels of VEGF. Moreover, administration of VEGF receptor inhibitor reduced the levels of MMP-9 [44]. The agonists of PPARγ, RAR and RXR are suggested to act as inhibitory NRs in the context of angiogenesis via reduction of VEGFC production in brown adipose tissue [45]. Although active *all-trans* RA (ATRA), which is a strong pan RAR agonist, stimulates angiogenesis and induces production of VEGFA and VEGFR2 mRNA in human lung microvascular endothelial cells, endogenous RA signalling may regulate alveolar maintenance and repair [46]. Our data show that treatment with NEt-4IB, which is a selective RXR partial agonist, did not activate RARs and significantly suppressed the PPE-induced augmented VEGF levels in BAL fluid in both acute and late phase emphysema. These data suggest that a selective stimulation of RXR by NEt-4IB may have a therapeutic potential to attenuate the progression of airway remodelling by persistently suppressing VEGF levels. In addition, the therapeutic capability of NEt-4IB to improve the proteinase/anti-proteinase imbalance may result of suppressing VEGF production via NR signals.

Although the mechanism of RXR partial agonists in the progression of emphysema are not fully elucidated, NEt-4IB may play a critical role in controlling the accumulation and activation of neutrophils by suppressing IL-17, KC and CXCL5 and by regulating the activation of Th1 cells. There are some limitations of this study design. First, we did not measure lung volumes. Lm can be used as a surrogate for airspace size and contains both alveoli and alveolar ducts, however it was reported that there was a significant correlation between Lm and DI, and DI is a more sensitive parameter for parenchymal alternation than Lm in human lung [19]. In our study, the values of Lm, DI and Cst which we measured to evaluate emphysematous change of the lung were consistently attenuated by the treatment with NEt-4IB. Although we did not measure lung volumes, our data could evaluate the effects of NEt-4IB in a murine model of emphysema. Second, we could not apply the long-term CS-exposed emphysema model, because this systematic protocol was not approved by the institutional animal care and use committee of Okayama University Medical School.

## Conclusions

Our study demonstrated for the first time that treatment with the novel RXR partial agonist NEt-4IB holds therapeutic potential for the development of emphysema. The mechanism of this novel RXR partial agonist may be that it plays a crucial role in attenuating neutrophilic airway inflammation, correcting the proteinase/anti-proteinase imbalance, and improving insufficient anti-oxidant activities. Although additional studies will be required to demonstrate that NEt-4IB may partially and permissively affect heterodimeric partners such as LXR or PPARs, NEt-4IB could be a promising strategy for the treatment of emphysema.

## Additional files

Additional file 1: Treatment with the novel RXR partial agonist NEt-4IB (0.015%) suppresses PPE-induced emphysema and body weight loss without hepatomegaly. (A) Experimental protocol. (B) Lm values. (C) Representative photographs of H&E-stained lung tissue (magnification: X200) (a) PBS/vehicle, (b) PBS/NEt-4IB 0.015%, (c) PPE/vehicle, (d) PPE/NEt-4IB 0.005%, (e) PPE/NEt-4IB 0.015%, (f) PPE/NEt-4IB 0.05%. (D) The ratio of body weight change during experiment. (E) The liver weight to body weight ratio on day 14. The results for each group are expressed as the means ± SEM. This experiment was started with 8 mice in PBS/vehicle, PBS/NEt-4IB 0.015%, and 10 mice in PPE/vehicle, PPE/NEt-4IB 0.005%, PPE/NEt-4IB 0.015%, PPE/NEt-4IB 0.05%. No mice were died during the experimental period. * Significant differences (*P* < 0.05) between PBS/vehicle and PPE/vehicle. ** Significant differences (*P* < 0.05) between PPE/vehicle and PPE/NEt-4IB 0.005%, PPE/NEt-4IB 0.015% or PPE/NEt-4IB 0.05%. # Significant differences (*P* < 0.05) between PPE/vehicle and PPE/NEt-4IB 0.015% or PPE/NEt-4IB 0.05%. † Significant differences (*P* < 0.05) between PPE/NEt-4IB 0.05% and other groups. (PDF 6712 kb)

Additional file 2: Absolute body weight, liver weight, and the amount of feed intake containing NEt-4IB and Bexarotene. The results for each group are expressed as the means ± SEM. This experiment was started with 8 mice in PBS/vehicle and PBS/NEt-4IB 0.015%, and 10 mice in PPE/vehicle, PPE/NEt-4IB 0.005%, PPE/NEt-4IB 0.015%, PPE/NEt-4IB 0.05%. No mice were died during the experimental period. * Significant differences (*P* < 0.05) between PPE/NEt-4IB 0.05% and PBS/vehicle, PBS/NEt-4IB 0.015%, PPE/vehicle, PPE/NEt-4IB 0.005%, or PPE/NEt-4IB 0.015%. † Significant differences (*P* < 0.05) between PPE/Bexarotene 0.015% and other groups. (PDF 29 kb)

## Abbreviations

ALP: Alkaline phosphatase
ALT: Alanine aminotransferase
AMY: Amylase
AST: Aspartate transaminase
BAL: Bronchoalveolar lavage
BAP: Bio-anti-oxidant power
COPD: Chronic obstructive pulmonary disease
CS: Cigarette smoke
CSE: Cigarette smoke extract
Cst: Static lung compliance
CT: Computed tomography
CXCL5: C-X-C motif ligand 5
DI: Destructive index
DLCO: Diffusing capacity of the lung carbon monoxide
HED: Human equivalent dose
KC: Keratinocyte chemoattractant
L/BW: Liver weight to body weight ratio
LAP: Leucine aminopeptidase
Lm: Mean linear intercept
LXRs: Liver X receptors
MMP-9: Matrix metalloproteinase-9
NF-κB: Nuclear factor-κB
NRs: Nuclear receptors
PBS: Phosphate buffered saline
PPARs: Peroxisome proliferator-activated receptors
PPE: Porcine pancreas elastase
RARs: Retinoic acid receptors
RXRs: Retinoid X receptors
TG: Triglyceride
TIMP-1: Tissue inhibitor of metaroproteiase-1
TNF-α: Tumour necrosis factor-alpha
VDRs: Vitamin D receptors
