# Supplementary figures and images for: A retinoid X receptor partial agonist attenuates pulmonary emphysema and airway inflammation
Source: Respir Res. 2019 Jan 3;20:2. doi: 10.1186/s12931-018-0963-0 (PMC6318915; doi:10.1186/s12931-018-0963-0)

(A)

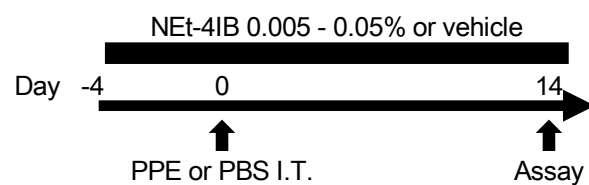

(B)

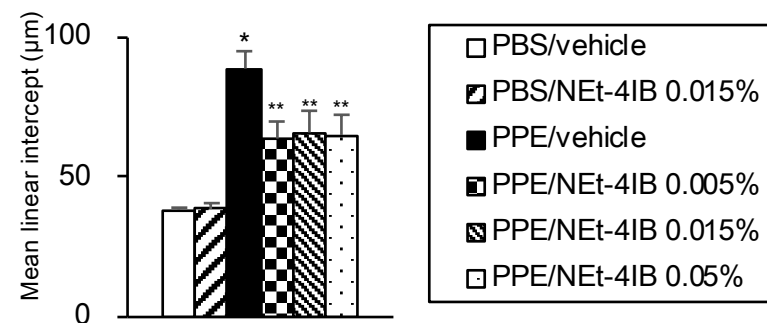

(C)

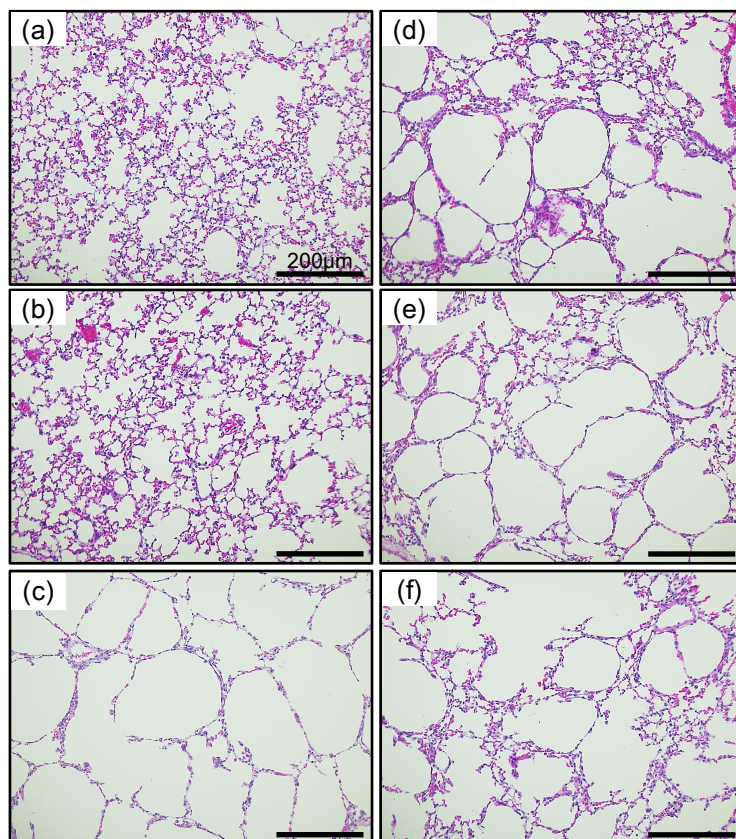

(D)

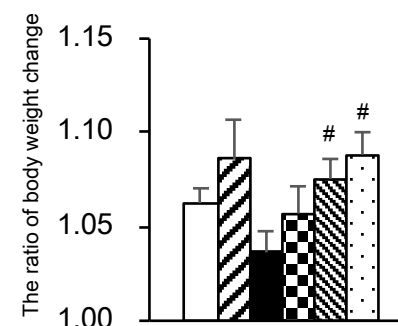

(E)

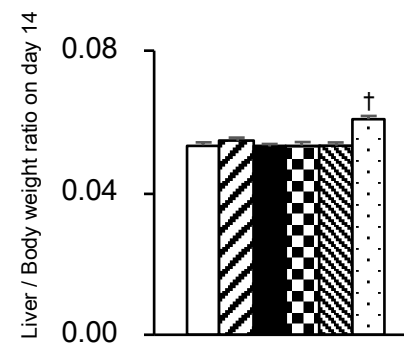

Supplement: Supplementary file 1 — Treatment with the novel RXR partial agonist NEt-4IB (0.015%) suppresses PPE-induced emphysema and body weight loss without hepatomegaly. (A) Experimental protocol. (B) Lm values. (C) Representative photographs of H&E-stained lung tissue (magnification: X200) (a) PBS/vehicle, (b) PBS/NEt-4IB 0.015%, (c) PPE/vehicle, (d) PPE/NEt-4IB 0.005%, (e) PPE/NEt-4IB 0.015%, (f) PPE/NEt-4IB 0.05%. (D) The ratio of body weight change during experiment. (E) The liver weight to body weight ratio on day 14. The results for each group are expressed as the means ± SEM. This experiment was started with 8 mice in PBS/vehicle, PBS/NEt-4IB 0.015%, and 10 mice in PPE/vehicle, PPE/NEt-4IB 0.005%, PPE/NEt-4IB 0.015%, PPE/NEt-4IB 0.05%. No mice were died during the experimental period. * Significant differences (P < 0.05) between PBS/vehicle and PPE/vehicle. ** Significant differences (P < 0.05) between PPE/vehicle and PPE/NEt-4IB 0.005%, PPE/NEt-4IB 0.015% or PPE/NEt-4IB 0.05%. # Significant differences (P < 0.05) between PPE/vehicle and PPE/NEt-4IB 0.015% or PPE/NEt-4IB 0.05%. † Significant differences (P < 0.05) between PPE/NEt-4IB 0.05% and other groups. (PDF 6712 kb) [file 12931_2018_963_MOESM1_ESM.pdf]
