# Supplementary material for: A retinoid X receptor partial agonist attenuates pulmonary emphysema and airway inflammation
Source: Respir Res. 2019 Jan 3;20:2. doi: 10.1186/s12931-018-0963-0 (PMC6318915; doi:10.1186/s12931-018-0963-0)
Supplement: Supplementary file 2 — Absolute body weight, liver weight, and the amount of feed intake containing NEt-4IB and Bexarotene. The results for each group are expressed as the means ± SEM. This experiment was started with 8 mice in PBS/vehicle and PBS/NEt-4IB 0.015%, and 10 mice in PPE/vehicle, PPE/NEt-4IB 0.005%, PPE/NEt-4IB 0.015%, PPE/NEt-4IB 0.05%. No mice were died during the experimental period. * Significant differences (P < 0.05) between PPE/NEt-4IB 0.05% and PBS/vehicle, PBS/NEt-4IB 0.015%, PPE/vehicle, PPE/NEt-4IB 0.005%, or PPE/NEt-4IB 0.015%. † Significant differences (P < 0.05) between PPE/Bexarotene 0.015% and other groups. (PDF 29 kb) [file 12931_2018_963_MOESM2_ESM.pdf]

Additional file 2

	PBS/vehicle	PBS/NEt-4IB 0.015%	PPE/vehcle	PPE/NEt-4IB 0.005%	PPE/NEt-4IB 0.015%	PPE/NEt-4IB 0.05%	PPE/Bexarotene 0.015%
BW on day 0 (g)	20.30 ± 0.37	20.19 ± 0.35	20.89 ± 0.28	20.16 ± 0.28	20.67 ± 0.34	20.54 ± 0.25	20.78 ± 0.30
BW on day 14 (g)	21.56 ± 0.31	21.89 ± 0.29	21.86 ± 0.18	21.29 ± 0.29	22.21 ± 0.28	22.34 ± 0.26 *	23.00 ± 0.23 †
Liver weight on day 14 (g)	1.15 ± 0.03	1.16 ± 0.02	1.15 ± 0.02	1.11 ± 0.03	1.14 ± 0.02	1.31 ± 0.02 *	1.67 ± 0.04 †
Feed intake (g/day/mouse)	2.87	2.69	2.73	2.78	2.74	2.81	2.79
RXR agonist intake (mg/day/mouse)	0	40.40	0	13.88	41.03	140.56	41.81
